# Effects of wettability on droplet movement in a V-shaped groove

**DOI:** 10.1038/s41598-018-34407-6

**Published:** 2018-10-30

**Authors:** Taeyang Han, Hyunwoo Noh, Hyun Sun Park, Moo Hwan Kim

**Affiliations:** 1 Division of Advanced Nuclear Engineering, POSTECH, Pohang, Gyeongbuk Republic of Korea; 2 Department of Mechanical Engineering, POSTECH, Pohang, Gyeongbuk Republic of Korea

## Abstract

As basic research to understand the behavior of droplets on structured surfaces, we investigated droplet movement in a V-shaped groove while the volume of the droplet changes. We developed a model to explain the mechanism of the droplet movement and the effects of the wettability of the inner walls of the groove on the droplet movement. Furthermore, the model predicted new phenomena and explains the effect of the nonhomogeneous wettability on droplet movement. The predictions of the model match the experimental results well. This research can provide the basic knowledge for manipulating droplets with structured surfaces for various applications.

## Introduction

Droplet manipulation to control the dynamic behavior or wetting modes of droplets is an essential technique for various applications such as increasing the efficiency of condensation heat transfer^1–4^, water–oil separation^5,6^, fabricating microlens arrays^7^, and energy harvesting by electrically-charged jumping droplets^8,9^. Initially, the droplet manipulation was achieved by chemical or thermal gradient methods^10–14^. Afterwards, natural phenomena such as directional movements of droplets on a lotus leaf^15^, wetted spider silk^16^ and cacti^17^ have demonstrated that the droplet manipulation can be achieved by geometric structures. Since then, many types of structured surfaces^5,6,18–23^ have been suggested for the droplet manipulation.

Effectiveness of the structured surfaces for the droplet manipulation has been proved by previous research. For example, the structured surfaces could increase the efficiency of condensation heat transfer^1–4^. When a droplet is on a hydrophobically coated structured surface (i.e., a superhydrophobic surface), the Cassie–Baxster^24^ state is energetically favorable, compared to the Wenzel^25^ state^26^. The adhesion energy between the droplet and the surface is minimized in the Cassie–Baxter state^27,28^. Therefore, the droplet, which acts as a thermal resistance in the condensation process, can be easily removed from the superhydrophobic surface^1–4,19,23,29–38^. However, when supersaturation exceeds a certain condition, the structures of the superhydrophobic surface become flooded. The flooding degrades the efficiency of the condensation heat transfer^39–41^. The reason of the flooding is that the adhesion energy can be increased when the number of the nucleated condensates increases, because nucleated condensates are generally pinned inside the structures. This problem will be solved, if the nucleated condensates can move out from the inside of the structures spontaneously. To induce the spontaneous movement, further understanding of the behavior of the droplets between the structures is necessary to design the appropriate structured surface.

In this study, the movement of a suspended droplet in a V-shaped groove was investigated to understand the behavior of droplets on structured surfaces. In fact, the droplet movement in the V-shaped groove was already studied in previous research. Initially, the effects of the capillary force on the droplet movement in the V-shaped groove has attracted attention owing to the particular feeding mechanism of shorebirds^42–45^. Then, artificial V-shaped grooves were created to mimic and analyze the particular droplet movement in a shorebird’s beak^46,47^. However, the mechanism and the effects of the wettability on the droplet movement were not sufficiently explained. To overcome these insufficiencies, we developed a model to explain the mechanism of the droplet movement and the effects of the wettability (the static contact angle (CA) and CA hysteresis) on the droplet movement in the V-shaped groove. Furthermore, the model expected new phenomena and explains the effect of the nonhomogeneous wettability on the droplet movement. To verify the suggested model, we conducted experiments, which observed the droplet movement in the V-shaped groove while the volume of the droplet changed.

## Modeling

**Figure 1:**
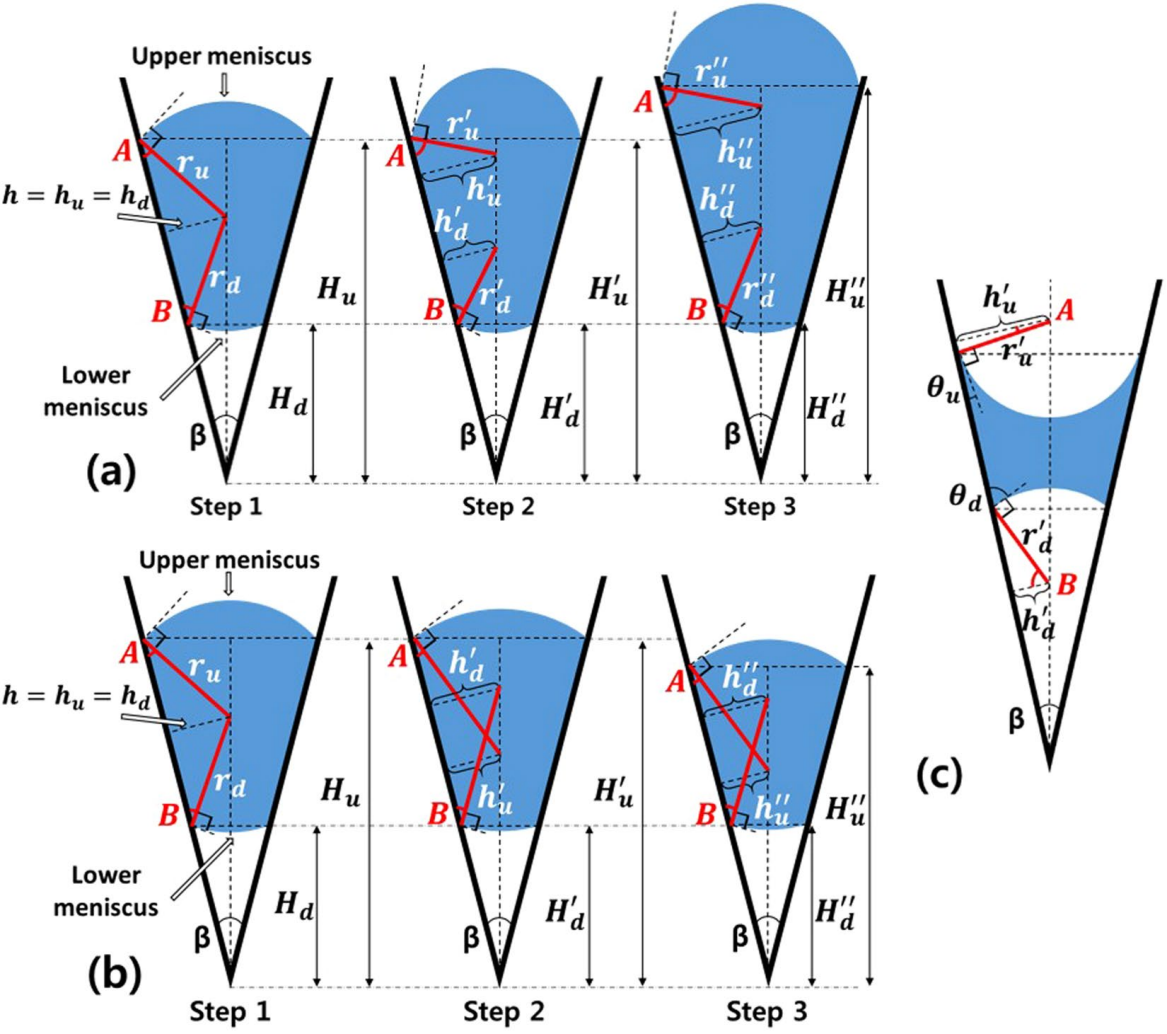
Schematic diagram of droplet movement in different wetting condition. (**a**) Droplet movement on the hydrophobic surface when V_drop_ increases. (H_u_ = H_u_’ < H_u_”) (**b**) Droplet movement on the hydrophobic surface when V_drop_ decreases. (H_u_ = H_u_’ > H_u_”) (**c**) A droplet on the hydrophilic surface. In (**a**,**b**), the contact angles of the upper and lower meniscus are equal to A + 90° and B + 90° respectively. In (**c**), the contact angles of the upper and lower meniscus are equal to A and B respectively.

In this study, the model that explains the movement of a suspended droplet in a V-shaped groove was created on the basis of three assumptions. First, we assumed that the droplet is symmetric with respect to the central plane in the V-shaped groove (Supplementary Fig. 1, X,Y plane). Because the normal force on the central plane can be neglected, we used a two-dimensional model to explain the behavior of the droplet in the V-shaped groove (Fig. 1). Second, the effect of the gravitational force on the droplet movement was neglected, because the radius of the droplet considered in this study (≤500 μm) is much less than the capillary length of water in the air (~2.7 mm)^26^. Third, the droplet movement considered in this study were assumed to be a quasi-equilibrium process. Thus, the pressure difference inside the droplet was neglected. In our experiment, we observed that the upper and lower menisci were changed from convex to concave simultaneously while the volume of the droplet changed (Supplementary Movie 1). It means that the flow induced by the pressured difference inside the droplet was very fast. Therefore, the assumption is reasonable to apply to this research.

The droplet in the V-shaped groove can be depicted as a cross-sectional diagram. The droplet is characterized by the CA (θ) of the inner wall of the V-shaped groove, the cross-sectional angle (β) of the groove, the radius (r) of curvature of the droplet and the height (H) of the meniscus of the droplet (Fig. 1). Subscripts u and d refer to the upper and lower menisci, respectively. When the droplet is suspended in the V-shaped groove, the CAs of the upper and lower menisci can be expressed as follows (Supplementary Info. 1):

1 $${\theta }_{u}=180-\frac{\beta }{2}-{\cos }^{-1}(\frac{{H}_{u}\,\tan (\frac{\beta }{2})}{{r}_{u}}),$$

2 $${\theta }_{d}=180+\frac{\beta }{2}-{\cos }^{-1}(\frac{{H}_{d}\,\tan (\frac{\beta }{2})}{{r}_{d}}).$$

The pressure difference between both sides of a curved interface is determined from the Young-Laplace equation, which is a function of the principal radii of curvature. In the experiment, the upper and lower parts of the droplet were exposed to the atmosphere, and we assumed that the pressure difference inside the droplet is negligible. Therefore, Equations (1,2) can be combined because the radii of the curvature of the upper and lower menisci are considered same, i.e., r_u_ = r_d_:

3 $${\theta }_{u}=180-\frac{\beta }{2}-{\cos }^{-1}(\frac{{H}_{u}}{{H}_{d}}\,\cos (180+\frac{\beta }{2}-{\theta }_{d}))$$

4 $${\theta }_{d}=180+\frac{\beta }{2}-{\cos }^{-1}(\frac{{H}_{d}}{{H}_{u}}\,\cos (180-\frac{\beta }{2}-{\theta }_{u}))$$

Equations (3 and 4) imply that θ_u_ and θ_d_ are related to each other and affected by H. This relationship can explain the mechanism of droplet movement.

## Results

### Driving force for droplet movement: effects of the static contact angle

θ_u_, θ_d_, H_u_ and H_d_ are related to each other (Eqs 3 and 4). This means that the movement of one meniscus can induce the movement of the other meniscus. Therefore, to understand the mechanism of the droplet movement, we should determine which meniscus moves first and in which direction. Fβconvenience, we call the meniscus that moves first the leading meniscus.

The motion of the leading meniscus is dependent on the static CA (θ_s_) of the inner walls of the V-shaped groove. Consider a droplet suspended on the hydrophobic or superhydrophobic inner walls of the groove. The radius of the curvature of the upper meniscus is expressed as

5 $${r}_{u}=\frac{{H}_{u}\,\tan (\frac{\beta }{2})}{\cos (180-\frac{\beta }{2}-{\theta }_{u})}.$$

Therefore, when a droplet is gently placed on the surface, the upper and lower menisci are convex, because θ_s_ > 90°. In addition, the droplet is almost spherical to minimize the surface energy^6^. Under these conditions, θ_u_ = θ_d_. Then, r_u_ and r_d_ share a center axis (Fig. 1a, Step 1). Once the volume (V_drop_) of the droplet increases, the droplet balloons; θ_u_ and θ_d_ increase. In this process, the radii of the curvature of both menisci shorten (r_u_ = r_d_ > r_u_’ = r_d_’). As a result, the center axes of the radii of the curvature of the upper and lower menisci become separated (Fig. 1a, Step 2). If we create right triangles that have r_u_ and r_d_ as the hypotenuses, h is defined as the height of the triangle (Fig. 1a). The heights (h_u_, h_d_) of the right triangles of the upper and lower menisci are equal in Step 1. However, in Step 2, h_u_ > h_d_. This means that the acute angle of the right triangle of the upper meniscus (A) is greater than the acute angle of the lower meniscus (B). Because θ_u_ > θ_d_ (; A + 90° = θ_u_, B + 90° = θ_d_), θ_u_ reaches the advancing CA earlier than θ_d_. Therefore, the upper meniscus is the leading meniscus and moves upwards first while V_drop_ increases (Fig. 1a, Step 3).

On the contrary, θ_u_ and θ_d_ decrease while V_drop_ decreases from Step 1. Then, r_u_ and r_d_ intersect each other because they lengthen (Fig. 1b, Step 2). If we construct right triangles (Fig. 1b) as done earlier, h_u_ is less than h_d_. This means that θ_u_ < θ_d_ (; A < B). Because θ_u_ reaches the receding CA earlier than θ_d_, the upper meniscus moves downwards first while V_drop_ decreases. Of course, if the hydrophobic surface has receding CA < 90°, the menisci can be changed from convex to concave while V_drop_ decreases (Supplementary Movie 3). When the curvature is concave, θ_u_ < θ_d_ (Fig. 1c). Therefore, the upper meniscus still moves downwards even though the curvature is inversed. The reason why θ_u_ < θ_d_ when the menisci is concave will be explained later.

**Figure 2:**
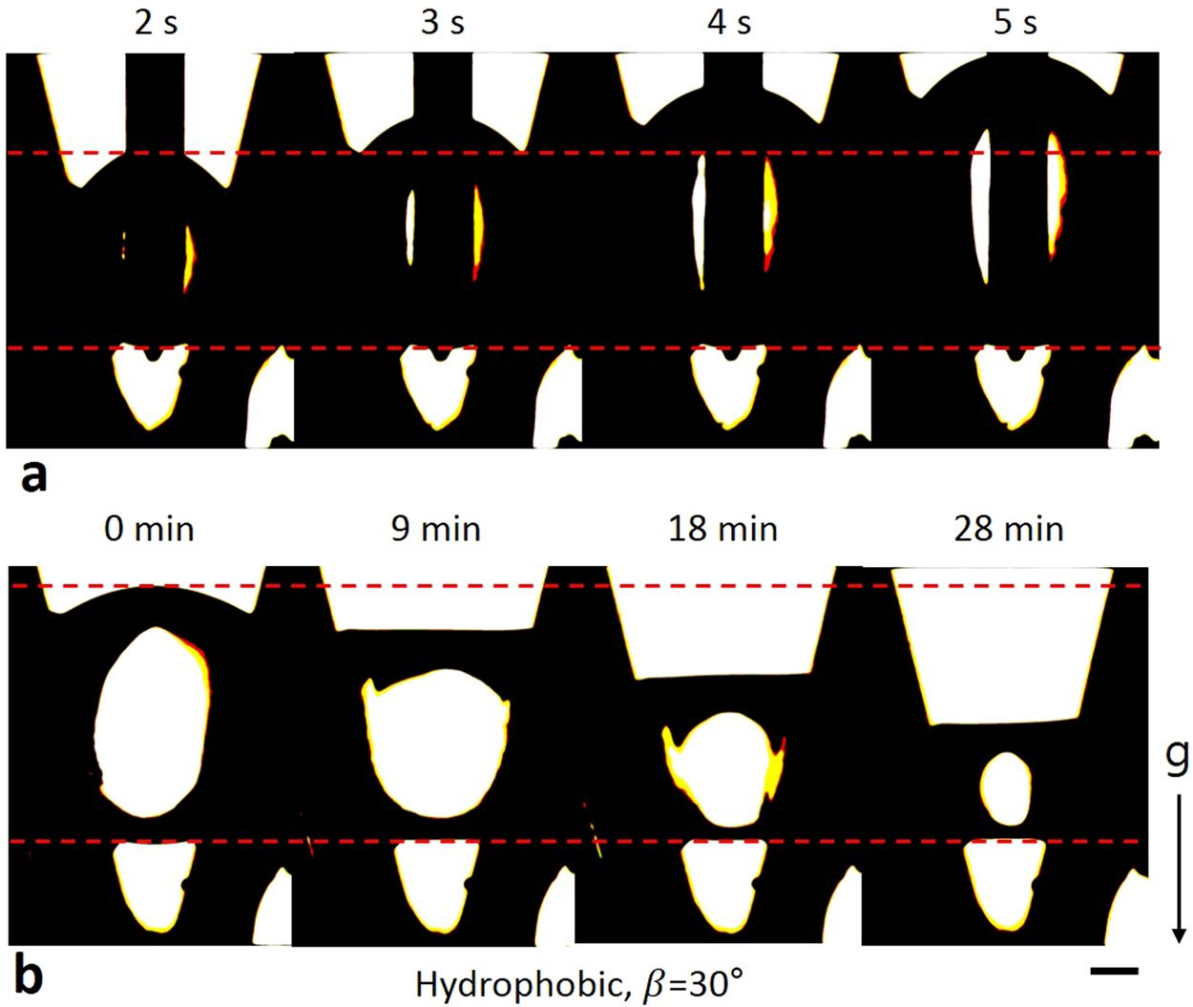
Droplet movement on hydrophobic surface. (**a**) The upper meniscus of the droplet moved upwards while V_drop_ increased. (**b**) The upper meniscus moved downwards while V_drop_ decreased. The lower meniscus was fixed in both cases. Marked time above images represent water injection and evaporation time respectively. Scale bar: 250 μm.

In summary, when the inner walls of the V-shaped groove are hydrophobic or superhydrophobic, the upper meniscus of the suspended droplet is the leading meniscus while V_drop_ changes. This expectation matches the experimental results from a previous study^6^ well. Present study also demonstrated this expectation experimentally. To verify the experiment, we used a same β as that of the previous research^6^, which is 30°. The observed trend of the droplet movement was identical to the previous result^6^. The leading meniscus (i.e. the upper meniscus) moved upwards and downwards while V_dorp_ increased and decreased respectively (Fig. 2, Supplementary Movies 2 and 3).

**Figure 3:**
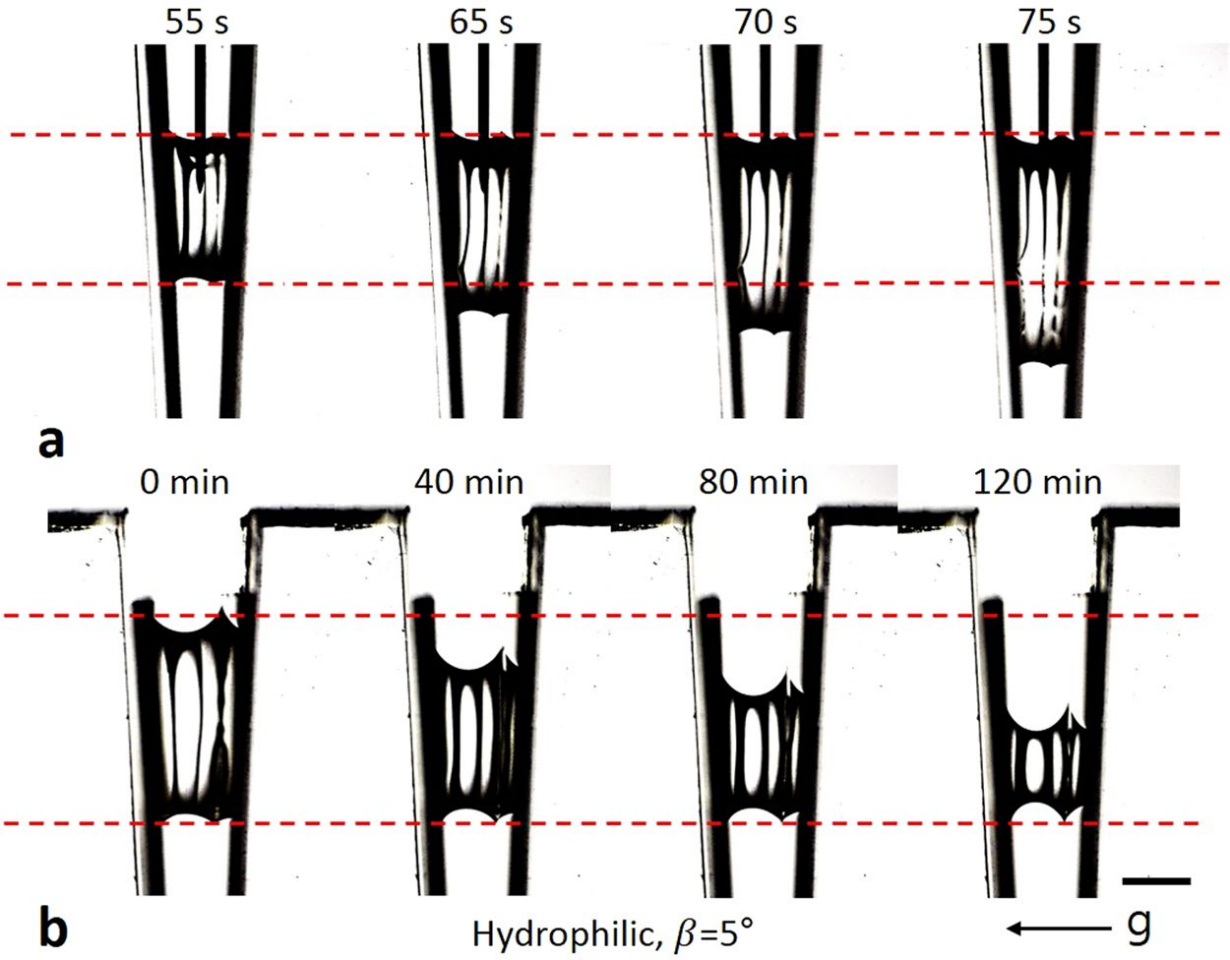
Droplet movement on hydrophilic surface. (**a**) The lower meniscus of the droplet moved downwards while V_drop_ increased. (**b**) The upper meniscus moved downwards while V_drop_ decreased. The leading meniscus moves downwards in both cases. The images are rotated 90° to the right (clockwise). Marked time above images represent water injection and evaporation time respectively. Scale bar: 2 mm.

If the inner walls are hydrophilic, a droplet can only be suspended on the groove when β is sufficiently low^6,45–47^. Therefore, the meniscus of the suspended droplet is generally concave because θ_s_ < 90° (Eq. 5). Then, r_u_ and r_d_ are outside the droplet (Fig. 1c); r_u_ and r_d_ never intersect. This means that the acute angle of the right triangle of the lower meniscus (B) is always larger than that of the upper meniscus (A). Because A = θ_u_ and B = θ_d_, θ_d_ is always larger than θ_u_ when the curvature is concave. In this condition, the leading meniscus is determined according to the process. When V_drop_ increases, the droplet balloons. As a result, θ_d_ reaches the advancing CA earlier than θ_u_. Then, the lower meniscus moves downwards first. In contrast, when V_drop_ decreases, the CAs of both menisci decrease. Thus, θ_u_ reaches the receding CA earlier than θ_d_. Then, the upper meniscus moves downwards first. In this situation, the lower meniscus gradually becomes more concave because the radius of curvature shortens while the upper meniscus moves downward (Eq. 5). Furthermore, CA of the lower meniscus decreases while the upper meniscus moves downward (Eq. 4). Therefore, the triple line of the lower meniscus moves upward or stays fixed. Finally, the both meniscus merge, and a liquid bridge formed in a groove breaks into two droplets. These phenomena were verified experimentally (Fig. 3, Supplementary Movies 4 and 5). In the movie 5, the lower meniscus seemed to move faster when the upper meniscus approached to the lower meniscus. It is because CA of the lower meniscus is changed faster when the distance between the both menisci is shorter (Eq. 4 and Supplementary Fig. 8). The characteristic of the droplet movement on the hydrophilic surface causes the spontaneous movement of a droplet on a shorebird’s beak; this phenomenon is called a “capillary ratchet”^42–45^. In this phenomenon, the droplet moves downwards by a tweezing motion of the groove (; β increases and decreases repeatedly) because the leading meniscus always moves downwards.

### Mechanism how the leading meniscus moves the other meniscus: effects of the contact-angle hysteresis

**Figure 4:**
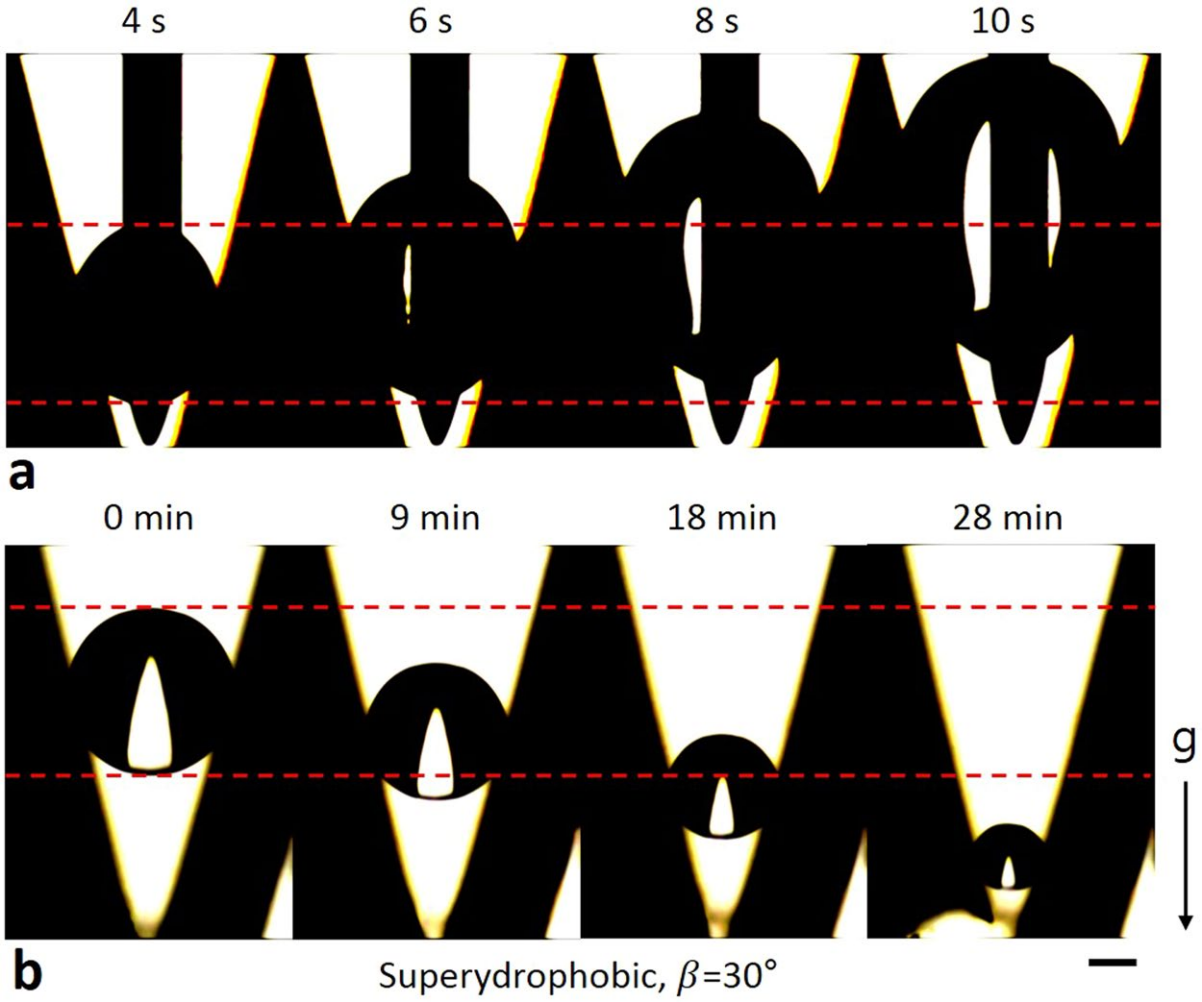
Droplet movement on superhydrophobic surface. (**a**) The upper meniscus of the droplet moved upwards while V_dorp_ increased. (**b**) The upper meniscus moved downwards while V_drop_ decreased. The lower meniscus moved in same direction with the upper meniscus. Marked time above images represent water injection and evaporation time respectively. Scale bar: 250 μm.

**Figure 5:**
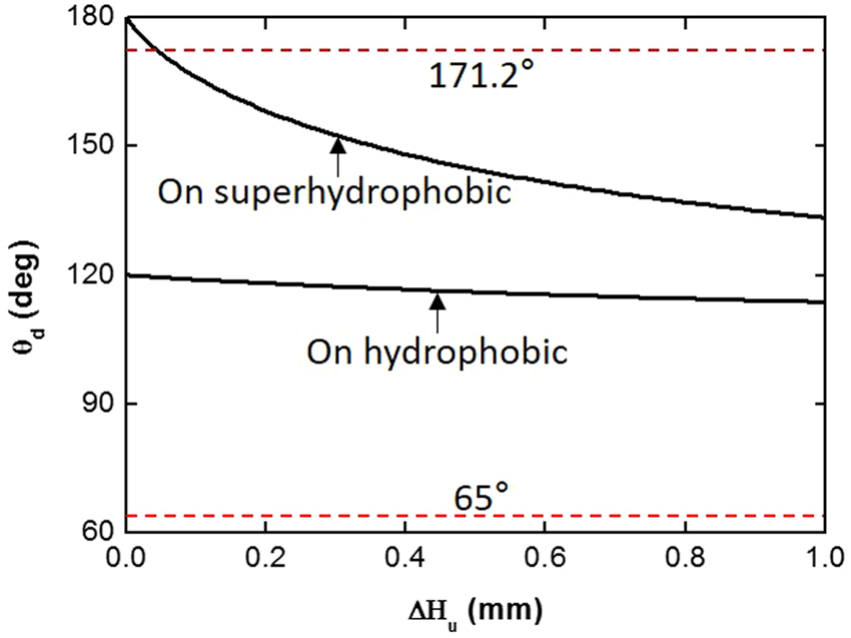
Contact angle of lower meniscus (θ_d_) Vs displacement of upper meniscus (ΔH_u_). Solid lines represent the decrease of the contact angle on the superhydrophobic and hydrophobic surface. Dashed lines mean the receding contact angles of a water droplet on the each surfaces. The receding contact angles on the superhydrophobic and hydrophobic surface are 171.2° and 65° respectively.

CA and H of the leading meniscus vary while the leading meniscus moves. As a result, CA of the other meniscus also varies (Eqs 3 and 4). This means that the motion of the leading meniscus can cause the other meniscus to move. In this process, the CA hysteresis plays a key role in the movement. We compared the movement of a droplet in V-shaped grooves with superhydrophobic and hydrophobic surfaces. When V_drop_ increases, the leading meniscus (i.e. the upper meniscus) moves upwards on the both type of surfaces (Figs 2a and 4a, Supplementary Movies 2 and 6). Then, the lower meniscus moves upwards on the superhydrophobic surface, whereas the lower meniscus stays fixed on the hydrophobic surface (Figs 2a and 4a). This phenomenon can be explained using Equation (4) and a schematic of droplet movement (Fig. 1). First, we consider the droplet movement when V_drop_ increases on the superhydrophobic surface. In this process, CA of the leading meniscus can be assumed to be equal to the advancing CA, 171.2°, because we consider slow processes such as condensation and evaporation. Then, β is a known value and H_d_ can be measured (; in our experiment, β = 30° and H_d_∼0.96 mm). It is obvious that H_u_ increases while the leading meniscus moves upwards (Fig. 1a, Step 3). Therefore, we can predict the change of θ_d_ while the leading meniscus moves. The result shows that θ_d_ decreases and reaches the receding CA due to the increase of H_u_ (Eq. 4, Fig. 5). It means that the lower meniscus can move upwards as a consequence of the upward movement of the upper meniscus on the superhydrophobic surface. However, the lower meniscus stays fixed on the hydrophobic surface, while the upper meniscus moves upwards. As done earlier, θ_d_ on the hydrophobic surface can be calculated using Equation (4). In this case, the CA of the upper meniscus can be assumed to be equal to the advancing CA on the hydrophobic surface, 120.3°. The result shows that θ_d_ on the hydrophobic surface also decreases while the upper meniscus moves upwards. However, θ_d_ cannot reach the receding CA, 65°, because the CA hysteresis is too large (Fig. 5). In conclusion, the CA hysteresis is the main reason to make the meniscus fixed. The analysis can be applied to the droplet movement while V_drop_ decreases (Figs 2b and 4b, Supplementary Movies 3 and 7).

### New phenomena expected from the model: effects of the nonhomogeneous wettability

#### Hybrid 1: Hydrophobic–superhydrophobic surface

**Figure 6:**
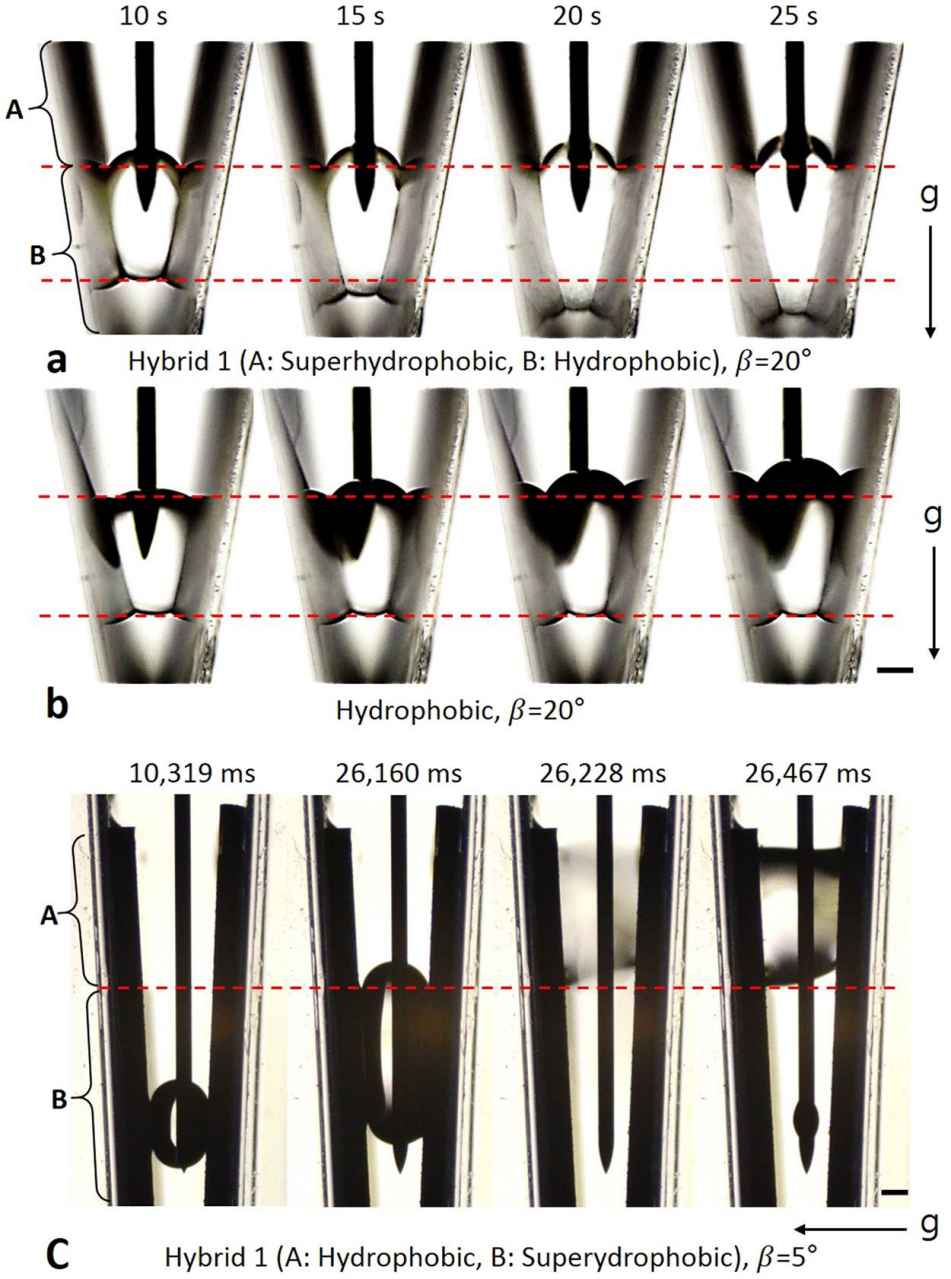
Droplet movement on hybrid 1 and hydrophobic surface while V_drop_ increases. (**a**) A Initially the droplet was on the hydrophobic area. Once the droplet reached the superhydrophobic area, the upper meniscus stayed fixed and the lower meniscus moved downwards on the hybrid 1 surface (See the triple line of the upper meniscus.). (**b**) The upper meniscus continuously moved upwards while V_drop_ increased on the hydrophobic surface. In both cases, water was injected at same position. Marked time above images means water injection time. (**c**) Initially the droplet was on the superhydrophobic area. Once the droplet contacted with the hydrophobic area, the droplet suddenly moved to the hydrophobic area. Scale bar: 500 μm.

When a droplet is gently placed on the hydrophobic surface in the groove (Fig. 1a, Step 1), the upper meniscus moves upwards as a leading meniscus while V_drop_ increases. However, if superhydrophobic area is above the upper meniscus, the Equation (3) predicts new phenomenon. When a 2-μL water droplet was on the groove having β = 20°, measurements determined that H_u_ ~2.8 and H_d_ ~1.7 mm. Then, the calculated value of θ_u_ is ~131° when θ_d_ reaches to the advancing CA of the hydrophobic surface, which is ~128°. It means that if the advancing CA of the superhydrophobic area is much greater than ~131°, the upper meniscus will stay fixed while the lower meniscus moves downwards. To verify this phenomenon, a surface that combines hydrophobic and superhydrophobic area was fabricated (Supplementary Fig. 3 and Info. 2), which is called hybrid 1. The advancing CA of the superhydrophobic area was ~177°. The hybrid 1 was installed in a groove; the lower part of the groove was hydrophobic, and the upper part of the groove was superhydrophobic. We selected β = 20°, which is smaller than that for the hydrophobic and superhydrophobic experiments. Using the small β is advantageous to have enough space below the lower meniscus, because a droplet is placed on the higher positon when β is smaller^41^. Water was injected to create a suspended droplet in the hydrophobic area of the hybrid 1. Then, V_drop_ was continuously increased. Once the upper meniscus reached the boundary of the superhydrophobic area, the upper meniscus could not move upwards. Instead, the lower meniscus continuously moved downwards (Supplementary Movie 8). To demonstrate the effects of the superhydrophobic area on the downward movement of the lower meniscus, the movements of droplets on the hybrid 1 and the hydrophobic surface were compared under the same conditions (Fig. 6a,b). Unlike the hybrid 1 case, the lower meniscus was pinned at a certain point when the inner wall of the groove was hydrophobic. Furthermore, in the hybrid 1 case, the upper meniscus ballooned when the lower meniscus descended in the groove. The reason for the ballooned meniscus is that θ_u_ increases because the radius of the curvature decreases when the lower meniscus moves downwards with a constant CA (Eq. 1).

On the other hand, when the hybrid 1 was installed reversely(superhydrophobic area was below the hydrophobic area), another phenomenon was observed. Initially the droplet was on the superhydrophobic area, and the droplet moved upward while V_drop_ increased. Once the upper meniscus contacted with the hydrophobic area, the droplet was suddenly drawn into the hydrophobic area (Fig. 6c, supplementary movie 12). This phenomenon is due to the drastic change of the radii of the curvature. θ_a_ of the superhydrophobic area is larger than that of the hydrophobic area. Therefore, the upper meniscus moves forward fast, when it contacts the hydrophobic area. The fast movement of the upper meniscus changes the radii of the curvature drastically. As a result, θ_d_ decreases and become smaller than θ_r_ of the superhydrophobic area.

#### Hybrid 2: Hydrophobic–superhydrophilic surface

**Figure 7:**
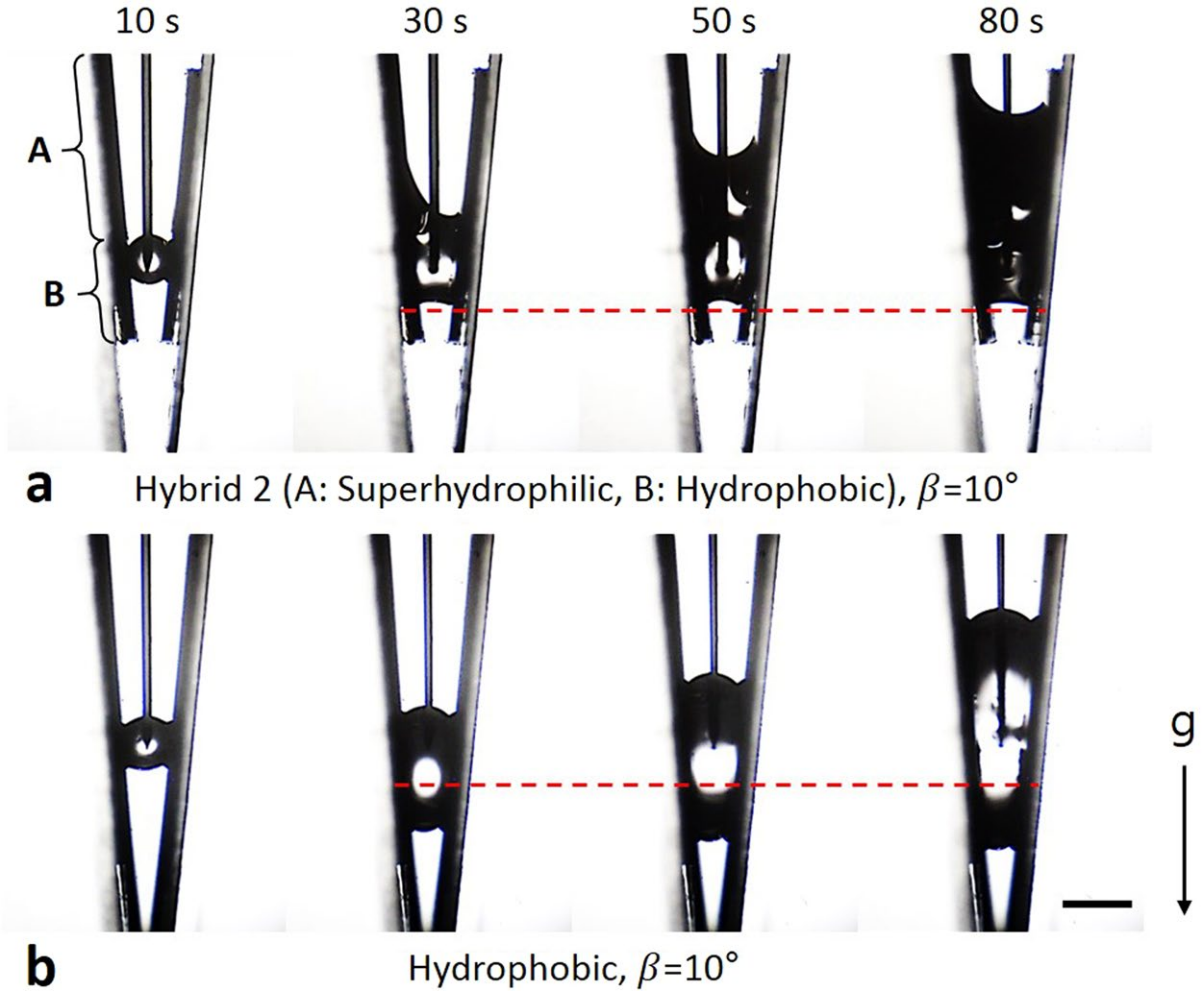
Droplet movement on hybrid 2 and hydrophobic surface while V_drop_ increases. (**a**) On the hybrid 2 surface, once the droplet reached the superhydrophilic area, the lower meniscus stayed fixed (30~80 s). (**b**) The lower meniscus continuously moved downwards while V_drop_ increased on the hydrophobic surface. In both cases, water was injected at same position. Marked time above images means water injection time. Dashed lines indicate the position of the lower meniscus on the hybrid 1 surfaces after 30 s. Scale bar: 2 mm.

When β ≤ 10°, we observed that the lower meniscus moved downwards while V_drop_ increased on the hydrophobic surface (Fig. 7b, Supplementary Movies 9 and 10). This result does not correspond with the expectation of the model, because of the gravity effect. For the cases of β ≤ 10°, we cannot put the needle deep into the groove because of a geometrical problem. As a result, the distance between the tip of the needle and the wall of the V-groove was larger when β ≤ 10°. The volume of an energetically stable droplet on a V-shaped groove increases when the length between the center position of the droplet and the wall (L; Supplementary Fig. 6) increases or β decreases (Supplementary Info.3 and Fig. 7). Since, the outlet of the needle (near the tip) can be considered as the center position of the droplet, the volume of the droplets in the cases of β ≤ 10° would be larger than that in the other cases. To verify the effect of the gravity, we calculated and compared the volume and the diameter of the droplets in each experimental cases (Supplementary Table 1). For the calculation, we assumed the curvature of the liquid-vapor interface is constant, because our model considered the energetically stable droplet as the initial stage of the droplet movement (Fig. 1(a,b) step 1). The result shows that only the diameter when β = 10° (the volume was ~17.5 μL and the diameter was ~3.5 mm) was large enough compared to the capillary length of water in the air (~2.7 mm)^26^. Therefore, when β≤10°, the menisci were drooped by the gravity and the lower meniscus moved downward. In this situation, we examine what will happens if the superhydrophilic area is above the upper meniscus of the droplet. Once the upper meniscus contacts the superhydrophilic area, the triple line of the upper meniscus rapidly moves upwards because the advancing CA of the superhydrophilic area is much less than that of the hydrophobic area. In this process, θ_u_ will reduce until it becomes less than the advancing CA of the superhydrophilic area. As a result, θ_d_ will decrease simultaneously (Eq. 4). Owing to the decrease of the CA of the lower meniscus, once the upper meniscus reaches the superhydrophilic area, the lower meniscus will no longer move downwards even though β is small enough.

To verify this phenomenon, a surface called hybrid 2 was prepared (Supplementary Fig. 3 and Info. 2). The hybrid 2 has a superhydrophilic upper part and a hydrophobic lower part. The hybrid 2 was installed in the V-shaped groove having β = 10°. A suspended droplet was generated in the hydrophobic area of the hybrid 2, and V_drop_ was continuously increased by the water injection. The upper meniscus moved upwards and finally reached the superhydrophilic area. Then, the radius of curvature instantaneously changed (Fig. 7a, Supplementary Movie 1). In this process, θ_d_ drastically decreased, as predicted by the model. As a result, the lower meniscus of the droplet on the hybrid 2 stayed fixed at a certain point, whereas the lower meniscus of the droplet on the hydrophobic surface continuously moved downwards (Fig. 7).

## Discussion

Previous studies suggested models^6,46,47^ to explain the movement of a droplet in a V-shaped groove. However, these models do not sufficiently explain the mechanism of droplet movement and the effects of the wettability. Firstly, the models^46,47^ to explain the mechanism of the droplet movement on a hydrophilic groove, which is called “the capillary ratchet” phenomenon, were suggested. One of the model^46^ mathematically proved that θ_d_ of the droplet in the groove is always greater than θ_u_. The other model^47^ explained “the capillary ratchet” phenomenon by comparing the pressures inside the menisci. These models gives an insight into understanding the mechanism of “the capillary ratchet” phenomenon. However, the models cannot be applicable when the inner walls of the groove are not hydrophilic. Secondly, an investigation^6^ of the droplet movement on the hydrophobic or superhydrophobic groove mathematically proved that the upper meniscus of the droplet always moves earlier than the lower meniscus while V_drop_ or β changed. The authors claimed that the movement of the upper meniscus could cause the movement of the lower meniscus. However, this research cannot logically explain the mechanism of the movement of the lower meniscus and the effect of the CA hysteresis on the movement.

In the present study, we explained the mechanism of the droplet movement in the various wettability condition. First, we explained the effect of θ_s_ on the movement of the leading meniscus, which acts as the driving force for the droplet movement. Second, the mechanism how the leading meniscus moves the other meniscus and the effect of the CA hysteresis on the movement of the other meniscus were explained. Furthermore, the model predicted the new phenomena and explained the effect of the nonhomogeneous wettability on the droplet movement. The predictions of the model match the experimental results well. In addition, this research only consider the droplet movement induced by the dynamic change of V. However, experimental results of previous research^6,46,47^ imply that β increase and decrease have same effect with V_drop_ decrease and increase respectively on the droplet movement. Therefore, our model can be applicable to explain the droplet movement induced by the dynamic change of β. This research can provide an insight how the wettability affects the droplet movement in the structured surface. It may help to manipulate droplets using structured surfaces for various applications. However, to predict the movement of a droplet in complicated structures, further studies that consider the three dimensional effect on droplet movement are needed.

## Method

### Overview

We conducted an experiment to observe the behavior of a droplet in a V-shaped groove. To make a droplet, a syringe pump system (Fusion 200) injected water between the inner walls of the V-shaped groove (Supplementary Fig. 4). In our experiment, the upper and lower menisci of the suspended droplet were exposed to the atmosphere. The droplet behavior induced by the change of V_drop_ was visualized using shadowgraphy. The experiment was conducted under indoor atmospheric conditions (room temperature: 21 °C, relative humidity (RH): 50%).

### Materials

We used an acrylic plate to create the V-shaped grooves. The acrylic plate had a thickness of 10 mm, and the V-shaped grooves were etched using laser-beam machining. We created three types of V-shaped grooves, which have β = 5, 10, 30° respectively.

To control the wettability of the inner walls of the V-shaped groove, we attached silicon wafers. The wettability of the silicon wafer was controlled by surface modification methods. A silicon wafer is naturally hydrophilic; thus, physical vapor deposition^2^ was used for hydrophobic functionalization. Normally, increasing the roughness of a surface emphasizes its wettability^26^. Therefore, we used a black silicon method^48^ to achieve superhydrophobicity and superhydrophilicity. Finally, hydrophilic, hydrophobic, superhydrophobic, Hybrid 1 (hydrophobic–superhydrophobic), and Hybrid 2 (hydrophobic–syperhydrophilic) surfaces were prepared. The wettability of the surface was characterized using a SMART DROP system (LM-6501RS), which measures the CAs (static CA θ_s_, advancing CA θ_a_, and receding CA θ_r_) of the samples (Table 1). However, the SMART DROP system could not measure the CAs on the superhydrophilic surface because water widely spread on the superhydrophilic surface.

**Table 1:** Contact angles of the surfaces.

Surfaces	Contact angle (deg)		
	θ_s_	θ_a_	θ_r_
Hydrophilic	49.4	53.1	25.2
Hydrophobic	111.5	120.3	65
Superhydrophobic	174.3	177.6	171.2

CAs of hydrophilic (bare silicon), hydrophobic (hydrophobically functionalized bare silicon) and superhydrophobic (hydrophobically functionalized-structured surface) respectively.

A DSLR camera (Nikon-D7000) with a resolution of 1920 × 1080 pixels and a frame rate of 24 fps recorded the droplet behavior.

### Experimental procedure

V_drop_ between the walls of the V-shaped groove was increased using a syringe pump system. A needle was connected to a syringe pump and positioned inside the groove. The syringe pump supplied water to the needle at a constant flow rate of ~0.45 μL/s. Therefore, a droplet was generated between the inner walls of the groove, and V_drop_ continuously increased. The decrease of V_drop_ was achieved by evaporation method.

The effects of the needle on the droplet movement is negligible, because the diameter of the needle (300 μm) is much smaller than that of the droplet and the CA of the needle is close to 90° ^6^. In addition, we experimentally verified the effect of the wettability of the needle on the droplet movement. We compared the movements of the upper meniscus on the superhydrophobic surface, when the needle was hydrophobically functionalized (Supplementary Fig. 5) or not. In both case, the movements of the upper meniscus were identical (Supplementary Movies 6 and 11).

## Electronic supplementary material

Supplementary movie 1

Supplementary movie 2

Supplementary movie 3

Supplementary movie 4

Supplementary movie 5

Supplementary movie 6

Supplementary movie 7

Supplementary movie 8

Supplementary movie 9

Supplementary movie 10

Supplementary movie 11

Supplementary movie 12

Supplementary material
