# Supplementary material for: Effects of wettability on droplet movement in a V-shaped groove
Source: Sci Rep. 2018 Oct 30;8:16013. doi: 10.1038/s41598-018-34407-6 (PMC6207755; doi:10.1038/s41598-018-34407-6)
Supplement: Supplementary file 13 — Supplementary material [file 41598_2018_34407_MOESM13_ESM.pdf]

Supplementary Information

Effects of wettability on droplet movement in a V-shaped groove

Taeyang Han, Hyunwoo Noh, Hyun Sun Park and Moo Hwan Kim

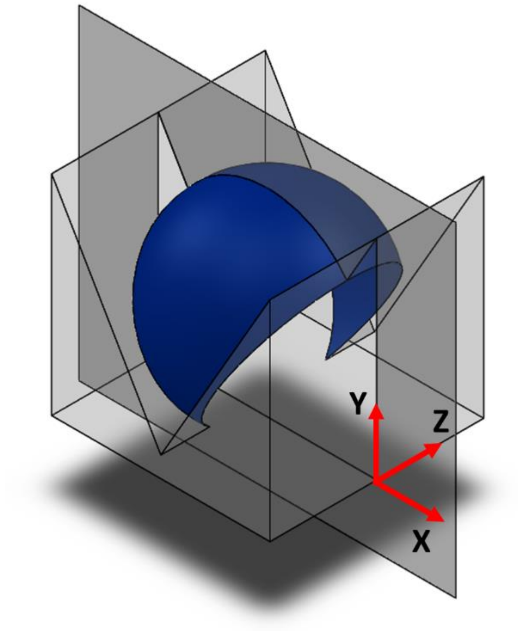

Supplementary Figure 1. 3D schematic diagram of a droplet on a V-shaped groove.

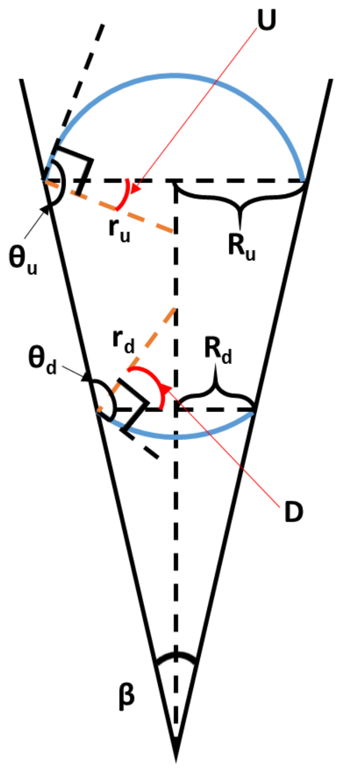

Supplementary Figure 2. 2D schematic diagram of a droplet on a V-shaped groove.

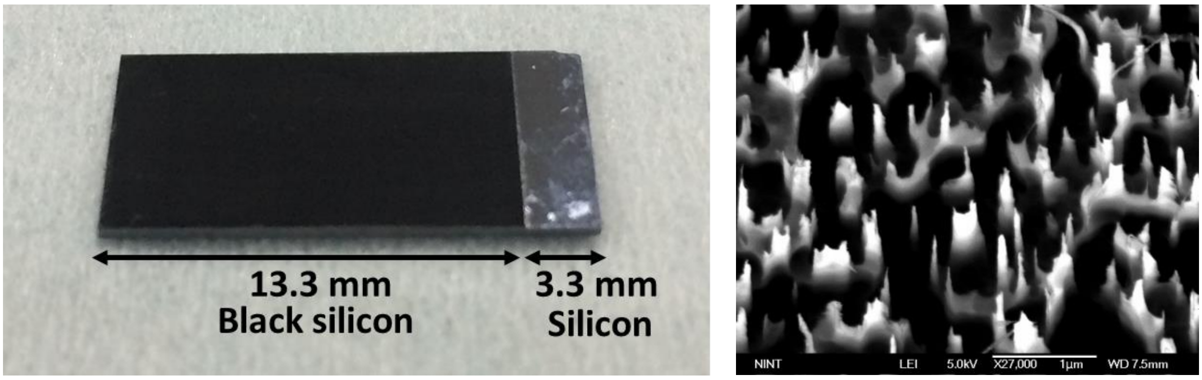

(a) (b)

Supplementary Figure 3. Geometry of hybrid surface. (a) Image of hybrid surface. (b) SEM image of black silicon area.

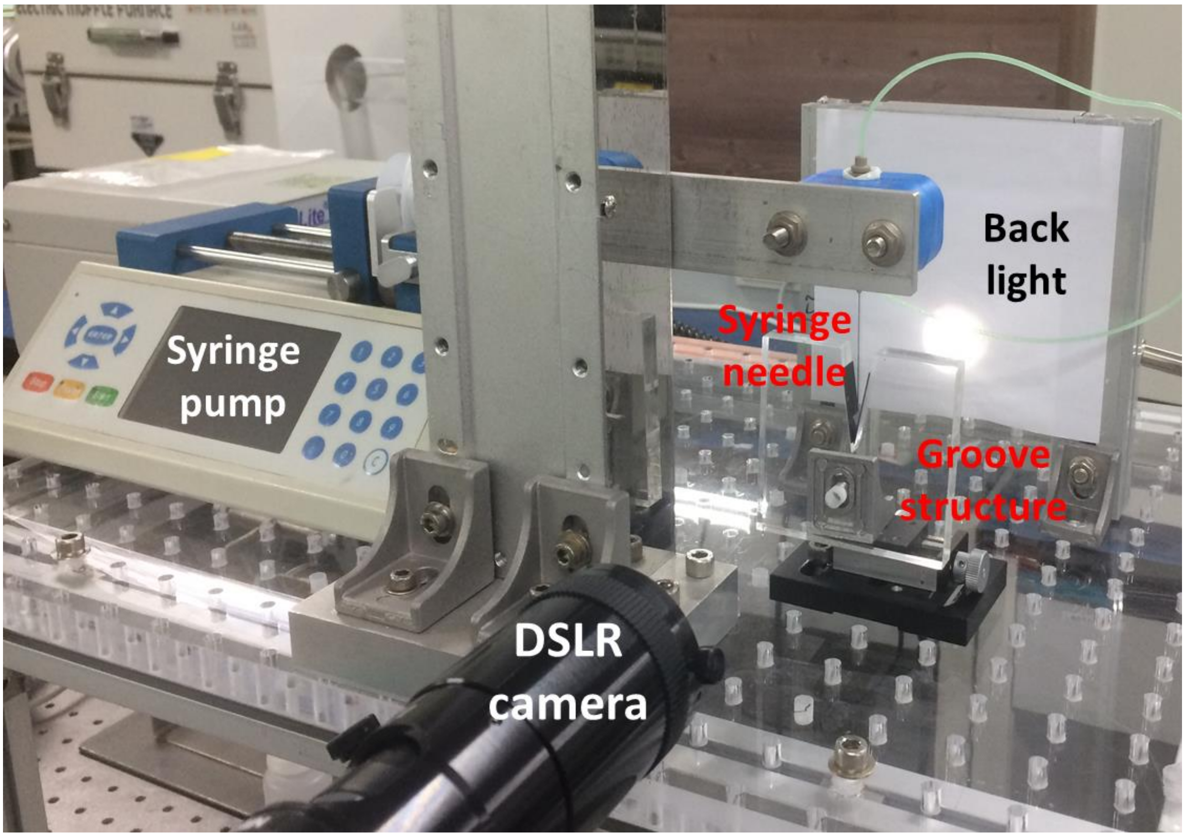

(a)

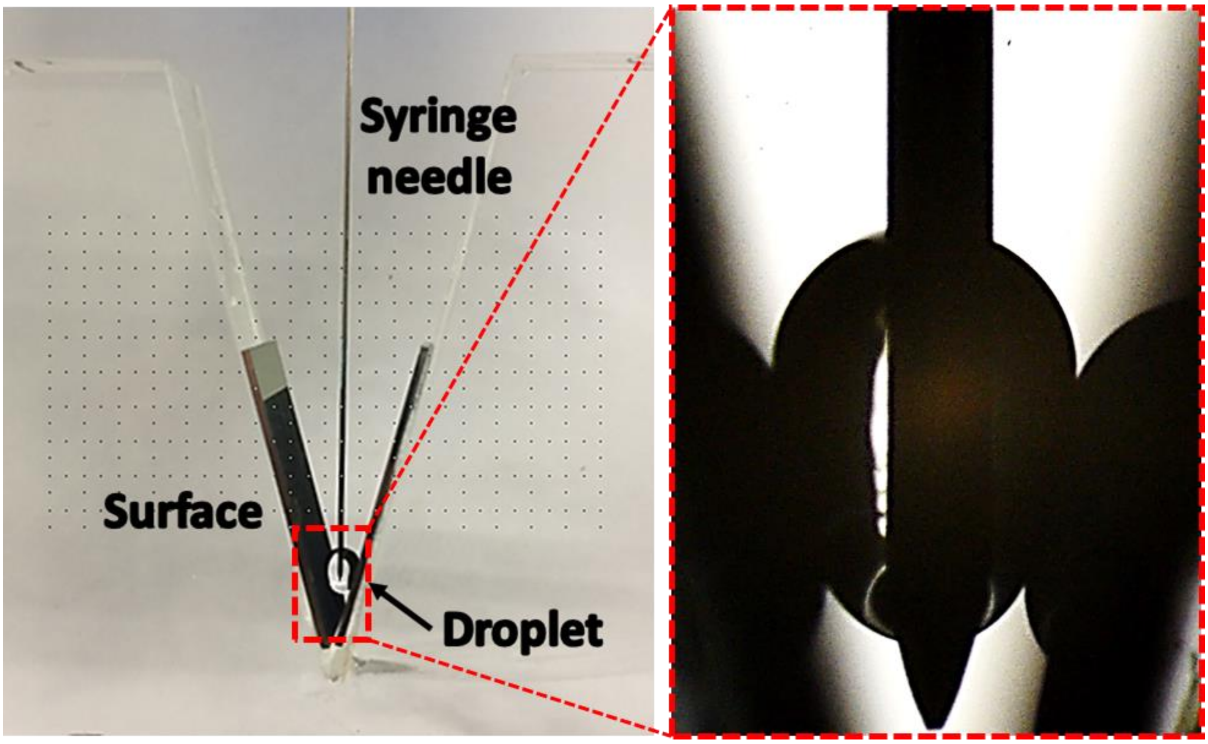

(b)

Supplementary Figure 4. Experimental apparatus. (a) Experimental system. (b) A droplet in the V-shaped groove.

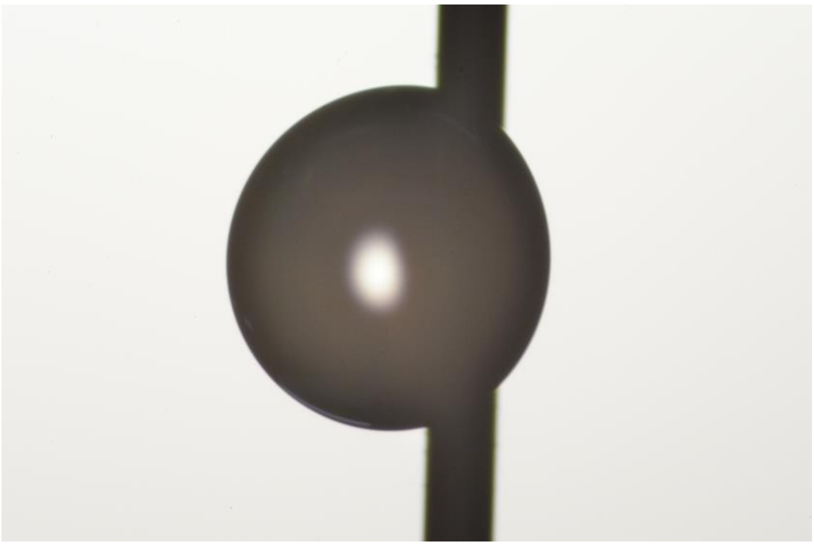

Supplementary Figure 5. A spherical droplet on the hydrophobically functionalized needle.

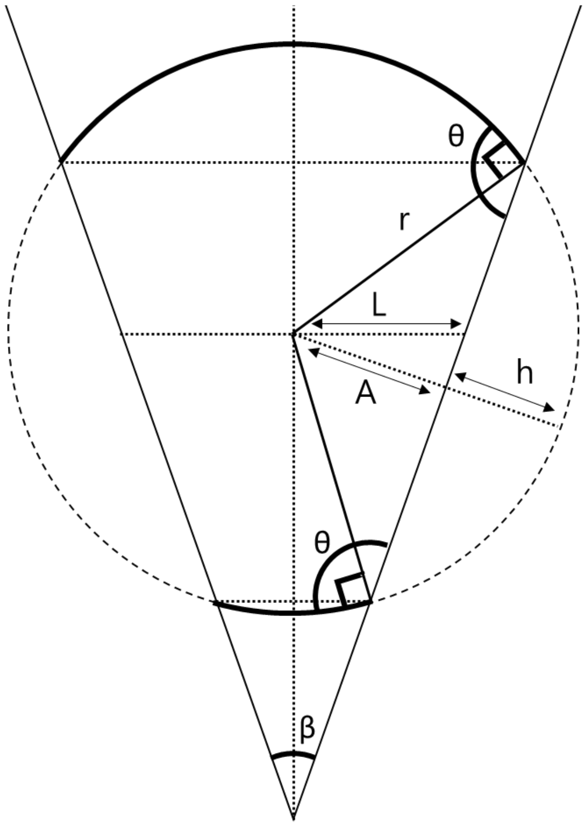

Supplementary Figure 6. 2-D schematic diagram of a stable droplet on a V-shaped groove.

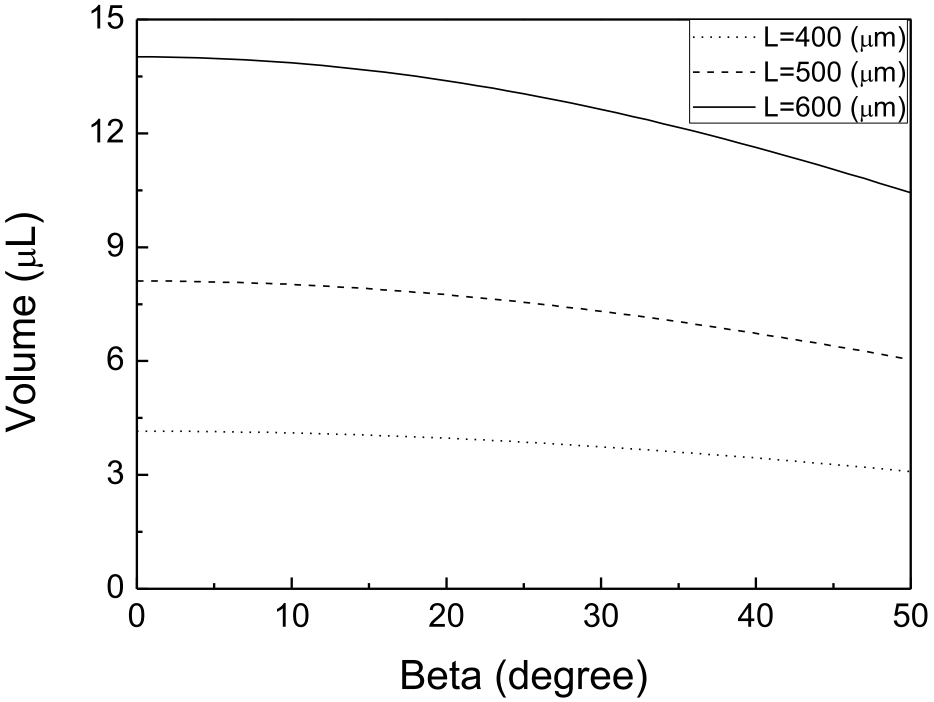

Supplementary Figure 7. Volume of a stable droplet on a V-shaped groove as function of β and L.

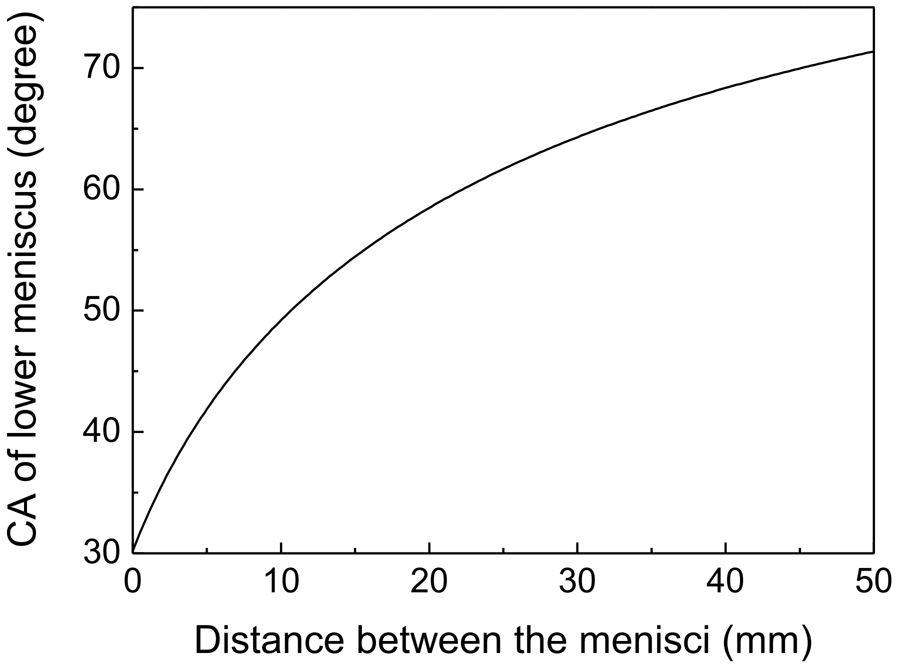

Supplementary Figure 8. Distance between the menisci Vs. CA of lower meniscus. This result is calculated from the Eq. (4) of the manuscript. Conditions of Fig.3 b experiment were used for the calculation. β=5°, θ_u=25.2° and H_d=34 mm.

Supplementary Table 1. Calculated volume (V) and diameter (D) of a energetically stable droplet on a V-shaped groove for each experimental cases.

	L (um)	Beta (degree)	V (ul)	D (mm)
**Fig. 2 (a)**	250	30	0.92	1.32
**Fig. 4 (a)**	250	30	0.92	1.32
**Fig. 6 (b)**	485	20	7.10	2.61
**Fig. 7 (b)**	650	10	17.65	3.54

Supplementary Information 1. Modeling of droplet movement in a V-shaped groove.

When X-Y plane is in the center of a droplet in a V-shaped groove, the droplet is symmetrical with respect to the plane (Supplementary Fig. 1). Therefore, 2D model was used to explain the droplet movement (Supplementary Fig. 2).

The contact angle of the upper meniscus can be derived by combining following equations.

$$r_u \cos U = R_u \quad (1)$$

$$U = 180 - \frac{\beta}{2} - \theta_u \quad (2)$$

Because $R_u = H_u \tan(\beta/2)$, the contact angle of the upper meniscus is expressed as follows.

$$\theta_u = 180 - \frac{\beta}{2} - \cos^{-1}(\frac{H_u \tan(\beta/2)}{r_u}) \tag{3}$$

The contact angle of the lower meniscus can be derived by combining following equations.

$$r_d \cos D = R_d \tag{4}$$

$$D = 180 + \frac{\beta}{2} - \theta_d \tag{5}$$

Because $R_u = H_u \tan(\beta/2)$, the contact angle of the lower meniscus is expressed as follows.

$$\theta_d = 180 + \frac{\beta}{2} - \cos^{-1}(\frac{H_d \tan(\beta/2)}{r_d}) \tag{6}$$

Supplementary Information 2. Characteristics of hybrid surfaces.

Hybrid 1 surface combines hydrophobic and superhydrophobic area. Hybrid 2 surface combines hydrophobic area and superhydrophilic area. The silicon area (Supplementary Fig. 3a) was coated using physical vapor deposition (PVD)[4], and used as the hydrophobic area in Hybrid 1 and 2. The black silicon area (Supplementary Fig. 3b) is naturally superhydrophilic, and turned to the superhydrophobic area using PVD. Therefore, the black silicon area was used as the superhydrophobic and superhydrophilic area in Hybrid 1 and Hybrid 2, respectively.

Supplementary Information 3. Characteristics of hybrid surfaces.

To figure out the reason why the lower meniscus moved downward, we made a model to calculate the volume of a droplet on the V-shaped groove. The curvature of the liquid-vapor interface is assumed as constant. From a 2-D schematic diagram (Supplementary Fig. 6), we derived equations to calculate the volume.

$$V = \frac{4}{3}\pi r^3 - \pi h^2 \left(r - \frac{1}{3}h\right) \tag{7}$$

$$h = r - A \tag{8}$$

$$A = L \cos\left(\frac{\beta}{2}\right) \tag{9}$$

$$r = \frac{A}{\sin(\theta - 90)} \tag{10}$$

The equations show that the volume is a function of distance between the center position of the droplet and the wall (L), static CA (θ) and the cross-sectional angle (β) of the groove. Then, the volume increases when L increases or β decreases (Supplementary Fig. 7).

Supplementary Movie 1. Droplet movement on the hybrid 2 surface while the volume of the droplet increases. The cross sectional angle of the groove was 10°.

Supplementary Movie 2. Droplet movement on the hydrophobic surface while the volume of the droplet increases. The cross sectional angle of the groove was 30°.

Supplementary Movie 3. Droplet movement on the hydrophobic surface while the volume of the droplet decreases. The cross sectional angle of the groove was 30°.

Supplementary Movie 4. Droplet movement on the hydrophilic surface while the volume of the droplet

increases. The cross sectional angle of the groove was 5°.

Supplementary Movie 5. Droplet movement on the hydrophilic surface while the volume of the droplet decreases. The cross sectional angle of the groove was 5°.

Supplementary Movie 6. Droplet movement on the superhydrophobic surface while the volume of the droplet increases. The cross sectional angle of the groove was 30°.

Supplementary Movie 7. Droplet movement on the superhydrophobic surface while the volume of the droplet decreases. The cross sectional angle of the groove was 30°.

Supplementary Movie 8. Droplet movement on the hybrid 1 surface while the volume of the droplet increases. The droplet was initially on the hydrophobic surface. The cross sectional angle of the groove was 20°.

Supplementary Movie 9. Droplet movement on the hydrophobic surface while the volume of the droplet increases. The cross sectional angle of the groove was 5°.

Supplementary Movie 10. Droplet movement on the hydrophobic surface while the volume of the droplet increases. The cross sectional angle of the groove was 10°.

Supplementary Movie 11. Droplet movement on the superhydrophobic surface while the volume of the droplet increases. The cross sectional angle of the groove was 30°. The needle was hydrophobically functionalized.

Supplementary Movie 12. Droplet movement on the hybrid 1 surface while the volume of the droplet increases. The droplet was initially on the superhydrophobic surface. The cross sectional angle of the groove was 5°.
